# An Adaptable Phase-Tracking System for Parkinsonian Rest Tremor: Design and In-Clinic Feasibility

**DOI:** 10.1109/JTEHM.2025.3625144

**Published:** 2025-10-24

**Authors:** Beatriz S. Arruda, Moaad Benjaber, John Fleming, Robert Toth, Colin G. McNamara, Andrew Sharott, Timothy Denison, Hayriye Cagnan

**Affiliations:** Department of Clinical and Movement NeurosciencesQueen Square Institute of Neurology, University College London London WC1N 3BG U.K.; Department of Engineering SciencesInstitute of Biomedical EngineeringUniversity of Oxford 6396 Oxford OX3 7DQ U.K.; MRC Brain Network Dynamics UnitNuffield Department of Clinical NeurosciencesUniversity of Oxford 6396 Oxford OX1 3TH U.K.; Amber Therapeutics Ltd. London W10 5YG U.K.; Department of PhysiologySchool of Medicine, College of Medicine and HealthUniversity College Cork Cork T12 CY82 Ireland; APC Microbiome IrelandUniversity College Cork Cork T12 CY82 Ireland; Department of BioengineeringImperial College London 4615 London W12 7TA U.K.

**Keywords:** Adaptive algorithm, electrical stimulation, Parkinson’s disease, phase estimation, precision medicine

## Abstract

Background: Tremor is the most common movement disorder and a prevalent symptom of neurodegenerative conditions such as Parkinson’s disease (PD). Given the limitations of medication, which may not effectively treat tremor, and the limited availability of surgical treatments such as deep brain stimulation, there is a pressing clinical need for non-invasive therapeutic alternatives, including peripheral electrical stimulation. The high variability of PD tremor poses a challenge to such therapies and calls for person-specific stimulation parameters. Methods: We developed a wrist-worn system incorporating an adaptable phase-tracking algorithm designed for real-time estimation of Parkinsonian rest tremor phase. The algorithm dynamically adapts to tremor variability, including changes in the axis of maximum excursion and center frequency. The system was first validated offline, followed by in-clinic feasibility testing in three individuals with PD. The system triggered the delivery of both phasic and open-loop electrical stimulation to the participant’s wrist. Results: Robust phase estimation was achieved both offline and in all participants. The system adapted to changes in tremor dominant axis and center frequency. Modest tremor modulation was observed at select person-specific settings. Conclusion: This work provides a novel platform for research involving tremor phase tracking, accounting for PD tremor variability, and a foundation for developing personalized, non-invasive tremor management strategies. Clinical and Translational Impact Statement—This study presents a wearable system for adaptive tremor phase tracking validated in individuals with Parkinson’s disease and establishes a foundation for further development of personalized non-invasive tremor management strategies. Category: Clinical Research

## I. Introduction

Tremor is the most common movement disorder and a dominant symptom of Parkinson’s disease (PD) [1]. PD, the second most common neurodegenerative disorder after Alzheimer’s disease, affects 0.3% of people worldwide and 1% of those aged above 60 years [2]. As the global population ages, it is expected that PD will become more prevalent [3]. Resting tremor affects over 75% of individuals with PD [4], [5], [6]. One of the most visibly noticeable traits of PD, tremor is often the first symptom that prompts individuals to seek medical attention [6].

Therapeutic interventions commonly employed in clinical practice include both pharmacological and surgical approaches. The most widely used pharmacological treatment is Levodopa, a dopamine precursor usually combined with Carbidopa to enhance central bioavailability [7], [8]. While dopaminergic medication can provide striking tremor relief, its effect on tremor is more variable and, on average, less consistent than its efficacy for other PD symptoms such as bradykinesia and rigidity [9], [10], [11]. Additionally, every PD medication is associated with side effects which may limit long-term use. Levodopa, for instance, can cause dyskinesias, nausea, and psychiatric symptoms [12], [13]. The state-of-the-art surgical approach is deep brain stimulation (DBS), which offers long-term improvement in motor function, including the alleviation of tremor symptoms [14]. However, due to the high cost and logistical challenges underlying invasive neurosurgery, less than 2% of eligible PD patients worldwide undergo DBS [15]. Therefore, there is a gap in the existing care for people with PD and a clinical need for non-invasive therapeutic interventions for tremor.

Peripheral electrical stimulation—interfacing at the wrist instead of directly with the brain—might disrupt tremor-related brain networks by evoking afferent activity, or alternatively, it could interfere with tremor signal transmission at the spinal level [16], [17], [18], [19], [20], [21]. Several related technologies have been developed and trialed for the treatment of essential tremor, including the Cala kIQ (Cala Health, Inc) wrist-worn device, which alternates high-frequency stimulation between the radial and median nerves [16], [17], [19], [21]. A clinical trial in people with essential tremor reported a 49% improvement in subject-rated tasks with this device, surpassing the 27% in a sham condition [19]. Subsequently, a larger home-use study found tremor suppression in 92% of users, with more than half experiencing at least a 50% reduction [17]. In individuals with PD action tremor, the device’s use resulted in a median tremor power reduction of 64%, with at least 50% reduction observed in 79% of users [16].

Recently, research into non-invasive essential tremor treatments has focused on stimulation phase optimization for personalized therapy. Phase-specific stimulation involves delivering electrical stimulation at targeted angles of the tremor cycle to modulate the underlying oscillatory activity [20], [22], [23]. Phasic stimulation at the median nerve has been shown to significantly modulate essential tremor, particularly considering its instantaneous changes [20]. Another study found that higher amplitude essential tremor was more effectively suppressed with higher amplitude, out-of-phase stimulation [18]. In PD, phase-optimized peripheral nerve stimulation was explored in a study using a large, non-portable pilot system to test the effects of phase-specific electrical stimulation at the median nerve [22]. This study highlighted the high variability of PD and revealed significant instantaneous changes in tremor severity when these distinct tremor oscillation patterns were considered within individuals. Depending on the person-specific stimulation phases, tremor could be significantly suppressed or amplified. However, as suggested by previous studies, the variability in tremor patterns posed challenges in accurate phase tracking due to shifts in the axis of maximum tremor excursion and in the tremor frequency with the highest power [22].

This study proposes a solution to these challenges: an adaptable phase tracking algorithm, integrated in a wrist-worn system and designed to account for tremor dynamic changes. Our wearable technology is the first of its kind to adapt to the changes in tremor dominant axis and center frequency when estimating phase. This paper describes the design and offline characterization of this system, followed by validation in a small cohort of people with PD resting tremor during in-clinic testing.

## II. Methods and Procedures

### A. Hardware

**FIGURE 1.**
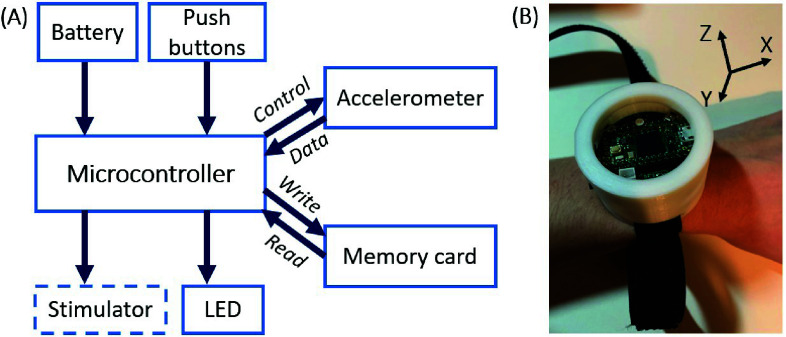
Hardware overview and assembly of the wrist-worn system. (A) Block diagram showing hardware components. The microprocessor board interfaces with a triaxial accelerometer, a memory chip, an LED, two push buttons, a battery, and a stimulator. The stimulator was kept externalized in this implementation. (B) The fully assembled system on a user’s wrist. The portable electronic components interfacing with the PCB were housed inside of a custom-designed enclosure. The three axes orientation for the on-board accelerometer is also depicted.

Hardware components were chosen to minimize the device size while ensuring sufficient memory, battery life, and processing power for accurate phase estimation and the execution of the planned experimental protocols. Key components, outlined in *Fig. 1A*, consisted of a compact, high-performance development board (Teensy 4.0, with an ARM Cortex-M7 processor clocked at 600 MHz), a triaxial accelerometer (Adafruit LSM6DSRTR), a 16 MB SPI flash memory chip (W25Q128JVSIQ), two push-buttons used to start or stop distinct stages of the experimental protocol upon a switch press, a red-green-blue light-emitting diode (LED) used to provide visual feedback to the experimenter on the protocol progression, a 3.7 V, 500 mAh lithium polymer battery capable of lasting 4 hours, and a circular-shaped custom printed circuit board (PCB) integrating all components. The hardware triggered stimulation via TTL pulses sent to a CE-marked stimulator, the only externalized element. Electronic components were housed inside a custom-designed and 3D-printed enclosure, as shown in *Fig. 1B*, which also illustrates the three axes orientation assumed in this paper. The fully assembled device weighed 48.7 grams, comprising the PCB (9.5 g), battery (8.8 g), and enclosure with wrist strap (30.4 g). The weight was deliberately minimized to avoid damping tremor amplitude during testing and falls within the range of typical commercially available wrist-worn devices [24].

### B. Software

The central component of the software consisted of an adapted version of the phase estimation algorithm *OscillTrack* [25], [26]. This algorithm was implemented offline in MATLAB (MathWorks, 2022b) for validation purposes, and online in C++, using the Arduino IDE (version 2.0.3). Our main contribution to the existing phase tracker was to add a self-tuning strategy which allowed the system to adapt to changes in the tremor axis of maximum excursion (dominant axis) and center frequency. Additionally, our system features included signal pre-processing in real-time (preceding its use by the phase-tracker); a method to calculate changes in tremor severity online (using an estimation of the tremor envelope); data saving to the memory card; as well as a user interface incorporating the push buttons, LEDs, and a command line interface (used for data retrieval). Data were sampled and stored at a rate of 208.03 Hz, the highest available in the accelerometer’s Arduino library and allowed by the memory constraints of our system. This sampling frequency surpassed both the Nyquist rate of 16 Hz and the minimum design requirement of 48 Hz for signals up to 8 Hz, allowing six equally spaced phases to be resolved per cycle.

The phase tracking algorithm followed a state-space model framework, which describes how a system evolves over time, accounting for its underlying dynamics. State-space models have two main elements: (i) an observation equation, representing the measured values of the system—encompassing, for example, noise—and (ii) a state equation, attempting to capture internal values of the system—for example, its instantaneous phase to be estimated [27], [28], [29]. The algorithm acts as a noise-driven harmonic oscillator: modelling tremor as a rhythmic signal distorted by noise, such as the variability in movement. It treats the underlying tremor as a pure sinewave and uses a mathematical framework to separate this theoretical signal from random fluctuations, allowing phase to be estimated in real time [27], [28], [29]. In practice, the state equation represents the real and imaginary components of the complex, analytic signals with each state variable corresponding to an oscillator at a fixed frequency. The state equation takes in the analytic signal and rotates it at that frequency, whereas the observation equation sums the real component of that signal, resulting in the prediction. From the rotating phasor corresponding to the real and imaginary components of the signal, we directly calculate the estimated phase and magnitude of an oscillation [29].

**FIGURE 2.**
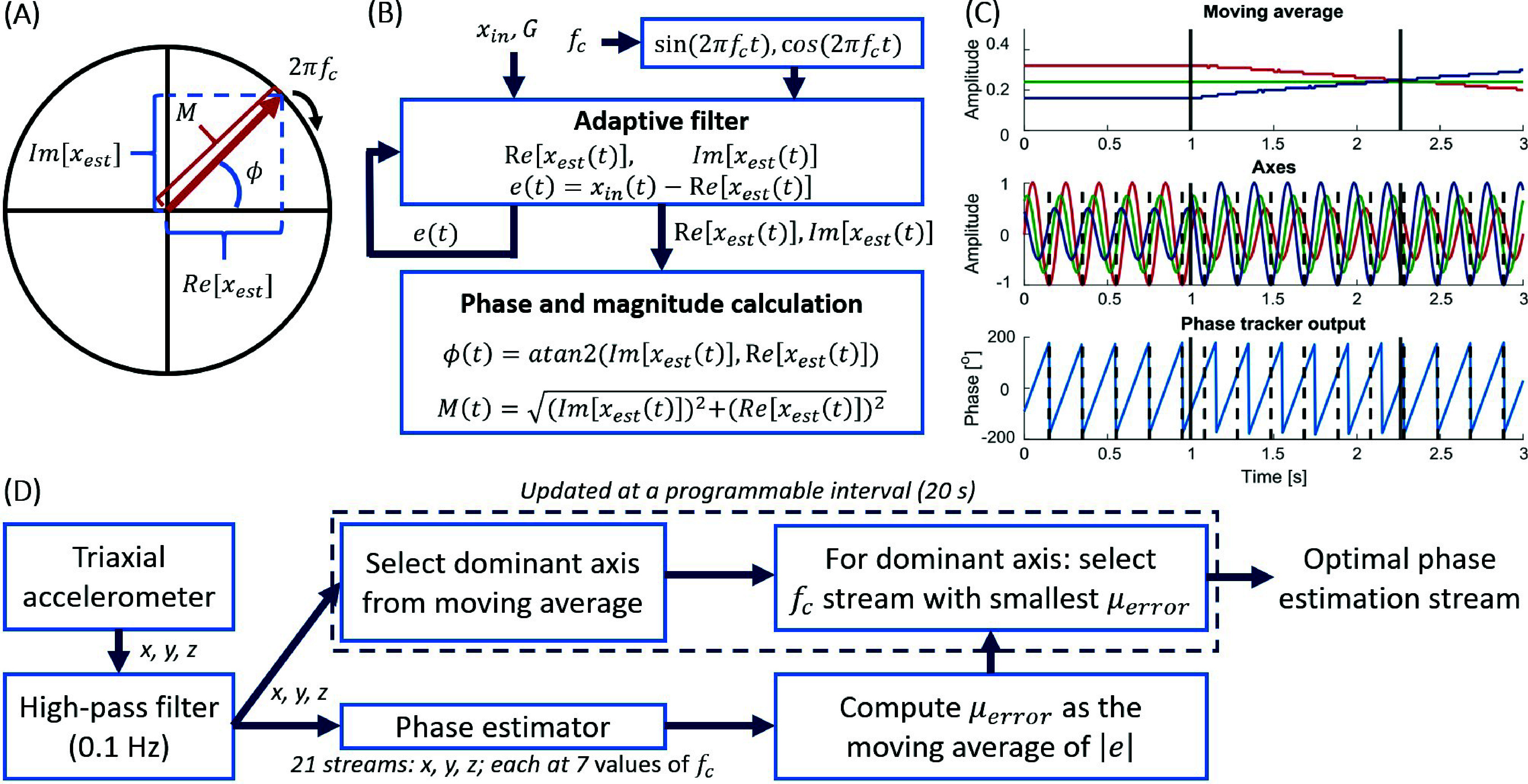
Phase estimation algorithm overview. (A) The rotating analytic signal in a two-dimensional space. The signal’s real component is represented along the horizontal axis and the imaginary component along the vertical axis. From those components, the phase ( $\Phi $) and magnitude ( $M$) can be calculated. (B) Phase tracking algorithm core operations. The input signal is compared to an estimate generated from the sine and cosine waves centered at the frequency of interest. The error, corresponding to the difference between these signals, adjusts the signal’s estimate. The phase and magnitude are obtained from the estimate’s real and imaginary components. (C) Dominant axis selection in example signals, where the dominant axis is selected according to the moving average of the absolute value of the signal amplitude. Example signals were pure sinewaves at 5 Hz with distinct amplitudes and phase shifts. (D) Summary of processing steps, including high-pass filtering for offset removal and an adaptive phase estimation strategy that accounts for varying dominant axes and center frequencies to select the optimal signal stream.

The algorithm thus represents the analytic signal ( $x_{est}$), with its real and imaginary components, as a vector rotating in the two-dimensional space at the reference frequency ( $f_{c}$) and defined by sine and cosine waves centered at that frequency (*Fig. 2A*). The real component of the analytic, estimated signal is recursively subtracted from the input signal ( $x_{in}$)—or the observed value—defining, for each time step ( $t$), an error term ( $e$), a measure of how much we can trust the model. An empirically derived real-valued constant between 0 and 1, $G$, recursively adjusts the signal estimate using $e$, and can be thought of as an analogue to the Kalman filter gain. A low $G$ reduces the error’s impact in adjusting the model whereas high $G$ increases the error’s ability to adjust the estimate. Thus, in a hypothetical scenario of high trust in the model—for example, if $x_{in}$ being tracked is a nearly sinusoidal wave with minimal noise— $G$ should be close to zero. Similar to the gain of a Kalman filter, the value of $G$ must be chosen to minimize the phase estimation error, which can be determined by comparing the phase estimated by the model to that derived from the gold-standard Hilbert transform. A variety of gain values was tested for Parkinsonian rest tremor as inverse powers of 2, and the phase estimates obtained with them were compared with the Hilbert-derived phases. Through this trial-and-error process, 0.25 was selected as the optimal choice, as it demonstrated the closest performance to the Hilbert transform.

The model operates as an adaptive band-pass filter whose width is defined by $G$ and should be chosen according to the noise levels of the system. As a result, $x_{in}$ does not need to be band-pass filtered a priori. The signal is high-pass filtered at a low cutoff frequency for offset removal prior to being input to the phase tracker. *Fig. 2B* summarizes the algorithm, with the input signal’s real and imaginary components, 90 degrees out of phase, representing the signal decomposition as a rotating phasor.

Signal pre-processing was required because accelerometer recordings typically present a DC shift, caused by gravitational acceleration as well as changes in temperature or in the device structure due to mechanical wear [30], and the phase-tracking algorithm assumes that $x_{in}$ is centered around zero. Consequently, if the signal has an offset, this will interfere with the model and the phase estimate. Before being input to the phase estimation algorithm, raw data from the triaxial accelerometer were high-pass filtered for offset removal with a high-pass SOS direct-form I second order Butterworth filter, with cutoff frequency at 0.1 Hz. Filter coefficients were generated using the MATLAB ‘filterDesigner’ tool.

For our wrist-worn device application, $x_{in}$ consisted of accelerometer-recorded Parkinsonian rest tremor oscillations. Those signals corresponded to the triaxial acceleration occurring as the user moved their arm in space. Tremor happens in three dimensions, and the dominant axis can fluctuate over time, thus the axis used as $x_{in}$ needed to be adjusted accordingly. Additionally, the tremor center frequency may also change and $f_{c}$ must adapt to that, in order to ensure that the model operates at the frequency closest to that of the signal. We accounted for the variability of tremor by implementing a self-tuning, adaptive strategy. This new component consisted of running in parallel various streams of the phase tracker, each of them corresponding to one combination of tremor axis, used as $x_{in}$, and $f_{c}$, within the target tremor signals’ band of interest.

Our implementation focused on Parkinsonian rest tremor, typically occurring from 3 to 7 Hz, with some accounts extending this range up to 8 Hz [5], [31]. To ensure comprehensive coverage and add a 1 Hz safety margin, we considered $f_{c}$ values within the 2 to 8 Hz range, with 1 Hz resolution, thus resulting in seven possible input frequencies. Those were run for each of the three axes (x, y, z), all high-pass filtered for offset removal, amounting to a total of 21 phase estimation streams continuously processed in parallel. One optimal phase tracking stream was chosen periodically at a programmable interval, accounting for the specifics of the testing protocol. In the intended tests with study participants, phase-locked stimulation was delivered in blocks, each of them at a single phase and lasting 10 seconds, preceded by a 10-second break without stimulation. Stream selection therefore occurred every 20 seconds, prior to the start of each 10-second stimulation block, and the chosen axis was kept throughout the block, to avoid switching the phase reference mid-block.

Before each stimulation block began, the dominant axis was selected as the one with the largest moving average, $\mu _{axis}$, of the absolute value of the accelerometer signal’s amplitude over the previous 10-second window, after offset removal. The moving average provided a low-pass filtered metric of the axis amplitude. *Fig. 2C* exemplifies the dominant axis selection based on the moving average for three test sinewaves, out of phase. The blue signal started with the largest amplitude, then it was overtaken by the red signal. Initially, the phase tracker continued considering the blue signal, but once the red signal’s moving average became the largest, the phase tracker adapted accordingly.

Similarly, $f_{c}$ was determined by first computing, for each of the seven frequency streams, the absolute value of $e$, the error signal corresponding to the difference between $x_{in}$ and $x_{est}$. The 10-second moving average ( $\mu _{error}$) was taken again, but then from the absolute value of this error. As the error reflected how well the estimated analytic signal represented the measured signal, the phase estimation stream with the smallest moving average corresponded to the one closest to the center frequency of $x_{in}$. For the axis already identified as dominant, $f_{c}$ was then selected as the frequency corresponding to the stream with the smallest $\mu _{error}$. Phase tracking was then locked to this stream and subsequently used for stimulation. Processing steps, including pre-processing and the adaptive strategy, are summarized in *Fig. 2D*.

Stimulation was triggered through TTL pulses sent from the development board to the stimulator. In the phasic stimulation state, this occurred when the phase estimate closely matched the target phase, and, once triggered, five pulses were delivered at 104.015 Hz (half the sampling rate). To avoid missed stimulation, it was permitted if either the current or the previous estimated phase matched the target phase. To mitigate the risk of missing the target phase due to phase resolution limits imposed by the sampling rate, stimulation triggering was also permitted if the tracked phase fell within an error budget centered around the target phase. This error budget was computed for each target center frequency according to the theoretical phase resolution, approximated as $f_{c}/f_{s} \cdot 360$ degrees, where $f_{s}$ is the sampling rate. Within our target $f_{c}$ values of 2 to 8 Hz and $f_{s}$ of 208.03 Hz, the error budget ranged from approximately 5 to 14 degrees.

### C. Matlab-Based Algorithm Characterization

We developed an offline version of the phase estimation algorithm in MATLAB to evaluate its performance and compare it with the C++ on-board version. The offline algorithm was tested using MATLAB-generated sinewaves (2 to 8 Hz) sampled at 208.03 Hz, matching the accelerometer’s sampling rate. Three conditions were tested: pure sinewaves, sinewaves with added noise followed by high-pass filtering, and sinewaves with added noise followed by band-pass filtering. To assess robustness, we subjected the algorithm to colored noise (brown and pink) to simulate real-world accelerometer noise [32], [33]. Phase estimates were compared to those obtained using a forward and backward second-order Butterworth filter from 1 to 9 Hz, followed by the Hilbert transform. Phase error was calculated as the absolute difference between the two methods, corrected for phase wrapping. This approach validated the algorithm’s accuracy and resilience to noise.

### D. In-Clinic Tests with People with Parkinsonian Rest Tremor

Finally, the device was validated in a cohort of three male study participants exhibiting Parkinsonian rest tremor, predominantly in their right hand. Tremor laterality was self-reported by participants and visually confirmed by the experimenter. All participants were treated with dopaminergic medication for their PD motor symptoms but omitted their last medication dose prior to the tests. Full medication regimens are detailed in *Table 4*. This study received approval from the Health and Social Care Research Ethics Committee A (HSC REC A) of the Health Research Authority, National Health Service (NHS, U.K.), in accordance with the Declaration of Helsinki (reference number: 19/NI/0009, date: 29/11/2021). All participants provided informed consent before participating in the tests. As part of the recruitment process, participants were screened to exclude individuals with a personal or family history of epilepsy or those with implantable devices.

**TABLE 1.** Phase error comparing the algorithm performance with the Hilbert transform (mean ± SD) [deg]

Frequency [Hz]	Error, pure signal	Error, band-pass filtered	Error, high-pass filtered
2	0.0 ± 0.3	1.4 ± 1.1	2.0 ± 1.7
3	0.0 ± 0.3	1.5 ± 1.1	1.8 ± 1.4
4	0.0 ± 0.2	1.5 ± 1.2	1.7 ± 1.3
5	0.0 ± 0.2	1.6 ± 1.3	1.8 ± 1.4
6	0.0 ± 0.2	1.8 ± 1.4	1.9 ± 1.5
7	0.0 ± 0.2	2.1 ± 1.6	2.2 ± 1.7
8	0.0 ± 0.2	2.7 ± 2.0	2.7 ± 2.1

**TABLE 2.** Phase errors comparing on-board and offline estimates for each study participant (mean ± SD) [deg]

ID	Between exact estimates	Between target and stim phases
P1	0.2 ± 0.5	4 ± 3
P2	2.4 ± 12.3	6 ± 5
P3	0.2 ± 0.3	6 ± 8

**TABLE 3.** Summary envelope metrics during distinct continuous stimulation segments (mean ± SD) [g]

ID	No stim (1 min)	Stim (final 1 min)	Stim (all 10 min)
P1	0.54 ± 0.11	0.29 ± 0.13	0.39 ± 0.12
P2	0.19 ± 0.10	0.37 ± 0.15	0.34 ± 0.19
P3	0.46 ± 0.21	0.46 ± 0.21	0.50 ± 0.21

**TABLE 4.** Participant medication profiles

ID	Medications
P1	Citalopram (20 mg, 1x/day), Sinemet Plus (25 mg, 1x/day), Tamsulosin (400 mg), Co-careldopa (25 mg), Ropinirole (6 mg, 1x/day)
P2	Ropinirole (12 mg, 1x/day), Madopar (12 mg, 3x/day)
P3	Levodopa (125 mg, 5x/day), Sinemet (125 mg at bedtime)

The wrist-worn system executed the experimental protocol, recording participants’ tremor using an on-board triaxial accelerometer (sampling rate of 208.03 Hz), estimated tremor phase online, triggered stimulation through the externalized CE-marked device (Digitimer Constant Current Stimulator DS7A), and saved data to a memory card. The externalized stimulator delivered pulses via a stainless-steel bar electrode attached to the device’s wrist strap.

Each study participant was instructed to sit on a chair, placing their arm over the chair’s armrest to allow unrestricted arm movement as their tremor emerged. Participants were directed to refrain from voluntary movements in their dominant tremor hand and to avoid actively suppressing their tremor. The hand was allowed to hang freely off the edge of the armrest in a relaxed, unsupported position, typically resulting in a neutral wrist orientation. This posture was selected by the participant as one that reliably elicited rest tremor and was visually confirmed by the experimenter prior to the start of the recording.

The device was placed on the wrist with the most prominent tremor, positioning the bar electrode over the median nerve on the wrist’s ventrum. Electrode paste was applied to reduce skin-electrode impedance. Correct electrode placement over the median nerve was verified by observing a visible thumb contraction and verbally confirming the participant’s perception of tingling during electrical stimulation. Stimulation during subsequent stages was capped at the highest level below the motor threshold that participants found comfortable. The experimental protocol lasted about 48 minutes and comprised two stages, each preceded by a baseline recording. The first stage, following a 10-minute baseline, was the random search, lasting approximately 27 minutes. Ten blocks, each with eight stimulation states, were presented. Prior to each block, the presentation order of the states was randomly shuffled without repetition to prevent confounding order effects [20], [22]. The eight stimulation states consisted of six phasic stimulation states from 0 to 300 degrees, spaced by 60 degrees, one open-loop, non-phasic setting, and sham (no stimulation). Each state was presented for 10 seconds, followed by 10 seconds of no stimulation. The second stage, presented after a 1-minute baseline, involved continuous stimulation for 10 minutes at the single stimulation state selected according to an on-board envelope approximation as the most suppressive.

The MATLAB (MathWorks, R2022b) data analysis involved (i) validating the phase estimator’s performance through comparison with its MATLAB-based offline version, (ii) examining the impact of stimulation during random search, and (iii) exploring the effects of continuous stimulation at the selected optimal state. The tremor envelope played a role in all analysis stages outlined above. The offline envelope was obtained by band-pass filtering (forward and backward, second-order Butterworth filter from 1 to 9 Hz) then applying the Hilbert transform to all accelerometer-recorded data points.

## III. Results

### A. Matlab-Based Algorithm Characterization

The algorithm demonstrated performance nearly identical to the Hilbert transform when applied to pure sinewaves, exhibiting a phase error close to zero. For signals that were either band- or high-pass filtered, the phase error ranged approximately between 1 to 3 degrees. For high-pass filtered signals, the mean error was no higher than 0.6 degrees in relation to that of band-pass filtered signals, underscoring the algorithm’s effectiveness without the need of a band-pass filter. *Table 1* shows the phase error (mean ± SD) for all tested oscillations.

### B. Algorithm Performance During In-Clinic Tests in People with Parkinsonian Rest Tremor

**FIGURE 3.**
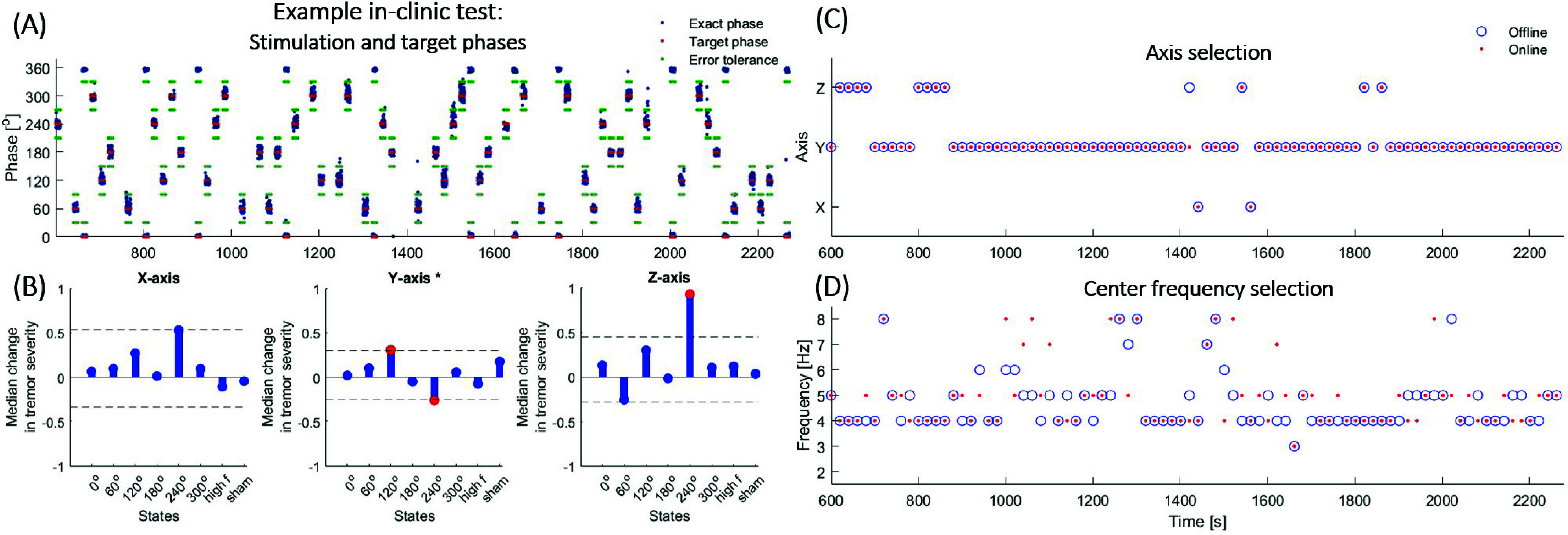
Example algorithm performance and random search stimulation effects for one participant (P3). (A) Exact (blue) and target (red) stimulation phases during the random search condition, showing close alignment within the 30-degree mean error limit (green). (B) Stimulation state-amplitude profiles showing the median change in tremor severity per state. The confidence limits generated with the Bonferroni correction for eight states are displayed as a dotted line, and the states for which the change in tremor severity was significant are indicated in red. The most frequent dominant axis, indicated in the plot title with ‘*’, was y. (C) On-board (blue) and offline (red) dominant axis selection updated every 20 seconds using accelerometer signal averages, with only one mismatch between methods. (D) On-board (blue) and offline (red) center frequency tracking updated every 20 seconds.

The mean overall phase error, comparing online and offline phase estimation, remained below 0.5 degrees for two study participants (labelled P1 and P3). P2, however, exhibited a slightly higher mean and standard deviation of the error, with the mean surpassing 2 degrees. This occurred because P2’s tremor stopped for long continuous segments, including for over one minute, and there is no accurate phase estimate without any oscillation. *Table 2* presents the mean and standard deviation of these phase errors, both the exact difference between online and offline estimates and, accounting for the error budget, between the target and actual stimulation phases. All values stayed well below the phase error tolerance of 30 degrees. *Fig. 3A* exemplifies the target and exact stimulation phases—encompassing the six anticipated target phases—in their presentation order, during the random search condition for one study participant (P3). In most data segments, the axis chosen by the on-board system matched the one selected by the offline version of the algorithm, thus confirming the reproducibility of the on-board axis selection, with a single mismatching segment likely due to rounding disparities as the moving average difference between the axes selected online and offline was less than 0.001 g (where 1 g corresponds to 9.81 m/ $\text{s}^{2}$).

Further analysis revealed clear differences in tremor behavior among participants, especially in terms of the consistency of tremor center frequencies. P1 displayed a remarkably consistent tremor, with the center frequency constantly selected at 4 Hz. In contrast, P2 and P3 exhibited more variability in their tremor center frequencies. When comparing the online center frequency with the median instantaneous frequency (computed offline with a 0.1 Hz resolution), the online frequency in P1 matched within 1 Hz in all cases. This was the case in 71% of segments for P2 and 81% for P3, indicating less consistency in their tremor patterns. The observed mismatches may be attributed to small inherent discrepancies between the C++ and MATLAB computations, possibly resulting from rounding differences, particularly when the moving averages of the error signal for competing frequency streams were very similar. \it{Fig. 3C-D} exemplifies the dominant axis and frequency selection, respectively, for P3.

### C. Random Search Stimulation Effects

We evaluated the stimulation state-amplitude profiles indicating the median change in tremor severity per state. *Fig. 3B* illustrates example profiles for P3, presenting the median change in tremor severity for all stimulation states and tremor axes. The data includes trials where the most frequently occurring dominant axis was tracked. The average number of trials per state used for generating Bonferroni-corrected confidence limits was eight for P1 and P3 and nine for P2. Considering that each bin corresponds to the median change in tremor for one of eight states, 37.5% of the total number of bins exhibited tremor suppression, while 62.5% showed tremor amplification. Significant tremor reduction was observed in two out of three study participants, P2 and P3. Specifically, P2 experienced 39% median tremor suppression at the constant, high-frequency (non-phasic) stimulation setting for the z (non-dominant) axis. On the other hand, P3 displayed 26% median tremor suppression at the 240-degree phasic stimulation setting for the y (dominant) axis. P3 also exhibited significant tremor increase, with 31% and 93% median tremor amplification occurring at the 120-degree setting for the y (dominant) axis and 240-degree for z (non-dominant), respectively. In the sham (no stimulation) condition, there was no significant change in tremor for any of the participants or their axes.

### D. Continuous Stimulation at the Most Suppressive State

The most suppressive random search states, selected for continuous stimulation, were constant high-frequency (open-loop, non-phasic), phasic at 180 degrees, and phasic at 240 degrees for P1, P2, and P3, respectively. *Table 3* summarises the mean and standard deviation of the most frequent dominant axis’s envelope in three segments: the 1-minute recording without stimulation, the final 1-minute of stimulation, and the entire 10 minutes of stimulation. For P2 and P3, individuals undergoing continuous phasic stimulation, the tremor envelope amplitude increased and remained close to that of the baseline recording when accounting for the standard deviation. Conversely, in P1, exposed to continuous non-phasic stimulation, tremor decreased compared to the baseline recording. The Wilcoxon rank-sum test was applied to compare the tremor envelope of the 1-minute baseline recording with that of the envelope during the final 1-minute of stimulation, focusing on the most frequent dominant axes. This analysis revealed significant differences across these segments in P1 and P2 (p $\ll$ 0.05 in both cases) but not in P3 (p = 0.2343).

## IV. Discussion

### A. Phase Estimation Algorithm Validation

The phase estimation algorithm has been validated as an effective tool for real-time phase tracking of Parkinsonian rest tremor, without requiring an a priori band-pass filter. Originally developed for tracking invasively recorded neural oscillations [26], the algorithm has been successfully adapted for use in a wrist-worn system designed to work with peripheral oscillations.

### B. Adaptive Strategy Assessment

The wrist-worn system demonstrated effective adaptability to changes in the dominant tremor axis, a feature that proved essential given the observed shifts in tremor orientation during the recordings, as depicted in Fig. 3C-D. The algorithm is capable of quick adjustment to changes in tremor dynamics, as demonstrated in *Fig. 2C*, which shows a transition to tracking a new dominant axis within 1.5 seconds. Our approach to dominant axis selection was straightforward, relying on a moving average calculation. An alternative approach, used in other studies, involves computing the tremor vector and estimating its phase [34]. However, we decided against this method due to several drawbacks: vector calculations can introduce artefacts in the signal, necessitate more intensive filtering, potentially add distortions arising from noise and non-linearities in individual axes, and may not capture the distinct tremor oscillation patterns that emerge, reflected by changes in the tremor dominant axis [22].

Regarding the selection of the center tremor frequency, considerable variability was noted in two of the three participants (P2 and P3). In these instances, the online frequency selection closely matched the offline criteria—including a replication of the algorithm and the median instantaneous frequency—within 1 Hz for at least 71% of the segments. P1, in turn, displayed a consistent center frequency. This consistency was mirrored in the frequency selection, with a perfect match between the online and offline data across all segments. This ability to adapt to tremor variability represents an advancement provided by our system and fills a gap left by previous studies, which did not account for such tremor fluctuations [22]. One important consideration is the observed concentration of segments around the 4 to 5 Hz frequency range. Future developments of this system may consider focusing the frequency selection more narrowly on this range, which could enhance the frequency resolution and better tailor the system to the most commonly observed tremor frequencies.

### C. Tremor Modulation Through Phasic and Open-Loop Stimulation

While our small cohort limits conclusions on therapeutic effects of stimulation, observed tremor suppression was modest. Only one out of three participants exhibited significant instantaneous tremor suppression at a phasic stimulation state. Tremor modulation achieved similar results as those of a pilot study exploring phasic stimulation in PD, which showed median significant tremor suppression of 36% and amplification of 117% [22].

Interestingly, in P3, who exhibited significant modulation under phasic settings, modest suppression of 26% was observed at the dominant (y) axis at the 240-degree setting. However, this was accompanied by a 93% amplification at the secondary (z) axis (*Fig. 3B*). Taken together with evidence for multiple rest tremor oscillation patterns—each potentially exhibiting distinct suppressive and amplifying phases—reported in earlier studies [22], this result suggests that effective suppression of one tremor mode through phasic stimulation may be accompanied by the emergence of another mode, potentially dominated by a different axis, even within the brief 10-second intervals used here to investigate instantaneous tremor changes. Such interactions between tremor modes may ultimately limit the efficacy of phasic stimulation as a therapeutic strategy.

Prolonged continuous phasic stimulation for 10 minutes did not conclusively suppress tremor in P2 and P3, while non-phasic continuous stimulation in P1 exhibited significant suppression—although non-phasic high-frequency stimulation in the random search condition did not produce significant instantaneous changes in tremor for any participant. In P2, the overall effect of phasic stimulation was amplification, and in P3, no significant effects of phasic stimulation were observed.

Phasic stimulation demands significantly greater computational resources compared to constant open-loop stimulation. This additional complexity involves real-time phase estimation, stimulation optimization for maximum suppression, and adaptive adjustments to detect and respond to shifts in tremor oscillation patterns. However, the observed effects of phasic and non-phasic peripheral nerve stimulation on tremor in this study did not reveal any substantial advantage of phasic over non-phasic stimulation. In contrast to the phase-specific stimulation explored in this study, the approach developed by Cala Health employs non-phasic, continuous stimulation exclusively [16], [17], [19], [21]. This open-loop strategy simplifies implementation and reduces computational resource requirements but may lack the adaptability needed to address dynamic tremor variability. The increased computational demands and the modest therapeutic benefits observed in this study suggest that the trade-offs between phasic and non-phasic approaches merit further investigation.

### D. Limitations and Challenges of This Study

First, our cohort was notably small, with only three participants. A larger cohort would provide a more robust basis for drawing conclusive observations regarding the potential therapeutic effects of phasic peripheral nerve stimulation on Parkinsonian rest tremor.

Additionally, the selected phase resolution of 60 degrees may be deemed insufficient to precisely capture the participant’s optimal suppressive stimulation phase. However, this resolution was a strategic choice aimed at minimizing the number of stimulation states to reduce the overall testing duration and thus prevent participant fatigue, especially considering the time spent in clinic. With a 60-degree resolution, our protocol allowed for the testing of eight stimulation states, including six phasic ones. The practical need to limit the duration of participants’ in-clinic sessions constrained the number of phases we could incorporate into the study. In future studies, particularly those employing a similar system in an ambulatory setting outside the clinic, different settings with finer resolutions could be explored.

The accelerometer’s wrist placement may also have limited the system’s ability to accurately capture changes in the dominant axis, thereby reducing the usage of the adaptive element in the wrist-worn system. With the sensor enclosed in a self-contained box on the wrist, as opposed to being isolated on the hand knuckle, the constraints on limb movements could lead to a bias in sensed oscillations, favoring sideways movement along the y-axis. While exploring alternative placements, such as incorporating the system into fingerless gloves, was considered, this was deemed less feasible for potential users, and the wrist placement was retained for practical reasons.

Another key challenge arises when tremor is suppressed during phasic stimulation, as the signal disappears and phase can no longer be tracked, yet stopping stimulation may cause the tremor to re-emerge. This scenario, which may often occur in real-world settings when rest tremor is suppressed at movement onset, requires further investigation.

Finally, this study focused exclusively on rest tremor, which facilitated signal characterization and feasibility testing in a controlled setting. While rest tremor is a hallmark of PD and occurs in over 75% of individuals with the condition, approximately 60% also experience tremor during action or movement [4], [5], [6]. A full clinical translation of this system would entail expanding its use beyond rest tremor to include tremor during movement and daily activities.

### E. Future Directions

A key next step is to investigate and compare the potential therapeutic effects of prolonged phasic versus non-phasic stimulation in suppressing tremor and to assess whether the additional computational demands of phasic stimulation are justified from a clinical perspective. This could involve prolonged application of the participant’s most suppressive stimulation setting—phasic or non-phasic—under controlled and real-world conditions. Based on the outcomes of these studies, adapting the system for at-home use as a medical treatment could be explored to enable its full translation into clinical practice.

Future studies should additionally explore strategies to manage periods when tremor is suppressed and phasic stimulation is not feasible. Potential approaches include continuing phasic stimulation using a sinewave at the last detected tremor center frequency, switching to open-loop stimulation, or discontinuing stimulation. The optimal solution may be person-specific, requiring further individual optimization. In real-world settings, users may also experience tremor during movement; thus, the feasibility and effectiveness of phase-specific peripheral nerve stimulation in suppressing action tremor should be investigated in future studies. Finally, to enable use outside controlled clinical environments, the system must achieve full portability. This includes integrating a self-contained, on-board stimulator safely embedded within the wrist-worn device, eliminating the need for external components and ensuring ease of use in everyday settings.

## V. Conclusion

In this study, we introduced and validated a wrist-worn device incorporating an adaptable phase estimation algorithm for tremor-like oscillations recorded by an accelerometer. The algorithm demonstrated robustness across offline and in-clinic settings. This system is the first to accurately track Parkinsonian rest tremor phase on-board while dynamically adapting to variations in tremor characteristics, such as center frequency and the axis of maximum excursion, addressing limitations identified in previous studies [22]. Our findings suggest that phasic stimulation may induce significant changes in tremor; however, the therapeutic efficacy of this technology and its potential advantages over non-phasic stimulation remain subjects of debate and invite further investigation. The custom-built device provides a platform to explore novel therapeutic strategies, such as phase-specific peripheral nerve stimulation, offering a pathway toward personalized, non-invasive treatment options for upper-limb pathological tremor. This study thus lays the groundwork for advancing both fundamental research and clinical investigations of wearable, phase-based peripheral stimulation for tremor management.

## APPENDIX Participant Medication Information

*Table 4* shows the medication regimens reported by each study participant. P1 also took Citalopram to manage depression, as listed. All participants withheld their last dose of dopaminergic medication before the study.

### Declaration of Interests

Colin G. McNamara and Andrew Sharott are inventors on a patent related to the subject matter of this article.
